# Combined deficiency of the Cnr1 and Cnr2 receptors protects against age‐related bone loss by osteoclast inhibition

**DOI:** 10.1111/acel.12638

**Published:** 2017-07-28

**Authors:** Antonia Sophocleous, Silvia Marino, Dilruba Kabir, Stuart H. Ralston, Aymen I. Idris

**Affiliations:** ^1^ Bone and Cancer Group Edinburgh Cancer Research Centre University of Edinburgh Crewe Road Edinburgh EH4 2XR UK; ^2^ Rheumatic Disease Unit MRC Institute of Genetics and Molecular Medicine University of Edinburgh Crewe Road Edinburgh EH4 2XU UK; ^3^ Department of Life Sciences School of Sciences European University Cyprus 6 Diogenes Street 1516 Nicosia Cyprus; ^4^ Department of Oncology and Metabolism Medical School Beech Hill Road Sheffield S10 2RX UK

**Keywords:** adipocyte, age, bone, Cannabinoid, Cnr1, Cnr2, osteoblast, osteoclast, osteoporosis

## Abstract

The endocannabinoid system plays a role in regulating bone mass and bone cell activity and inactivation of the type 1 (Cnr1) or type 2 (Cnr2) cannabinoid receptors influences peak bone mass and age‐related bone loss. As the Cnr1 and Cnr2 receptors have limited homology and are activated by different ligands, we have evaluated the effects of combined deficiency of Cnr1 and 2 receptors (Cnr1/2^−/−^) on bone development from birth to old age and studied ovariectomy induced bone loss in female mice. The Cnr1/2^−/−^ mice had accelerated bone accrual at birth when compared with wild type littermates, and by 3 months of age, they had higher trabecular bone mass. They were also significantly protected against ovariectomy‐induced bone loss due to a reduction in osteoclast number. The Cnr1/2^−/−^ mice had reduced age‐related bone loss when compared with wild‐type due to a reduction in osteoclast number. Although bone formation was reduced and bone marrow adiposity increased in Cnr1/2^−/−^ mice, the osteoclast defect outweighed the reduction in bone formation causing preservation of bone mass with aging. This contrasts with the situation previously reported in mice with inactivation of the Cnr1 or Cnr2 receptors individually where aged‐related bone loss was greater than in wild‐type. We conclude that the Cnr1 and Cnr2 receptors have overlapping but nonredundant roles in regulating osteoclast and osteoblast activities. These observations indicate that combined inhibition of Cnr1 and Cnr2 receptors may be beneficial in preventing age‐related bone loss, whereas blockade of individual receptors may be detrimental.

## Introduction

Endogenous cannabinoid (endocannabinoid) ligands and their receptors play important roles in the regulation of bone mineral density in animal models of bone disease (Bab *et al*., 2009; Idris & Ralston, 2012) and in human (Sophocleous *et al*., 2017). The endocannabinoid system comprises two known receptors, a family of endogenous ligands and various enzymes that are responsible for ligand synthesis, transport and inactivation. Endocannabinoid ligands and their receptors regulate a wide variety of neurological functions including appetite control, pain perception, motor function and the immune response (Matsuda *et al*., 1990; Idris & Ralston, 2010). There are two classic cannabinoid receptors: type 1 (Cnr1) and type 2 (Cnr2) receptors, both of which belong to the G protein‐coupled receptor (GPCR) super‐family (Pertwee & Ross, 2002; Demuth & Molleman, 2006). The Cnr1 receptor is strongly expressed in the nervous system (Matsuda *et al*., 1990; Idris & Ralston, 2010), whereas Cnr2 is predominately expressed in cells of the immune system (Munro *et al*., 1993). However, both receptors are also expressed in bone cells including osteoclasts, osteoblasts, osteocytes and chondrocytes (Tam *et al*., 2006, 2008; Idris *et al*., 2009; Idris & Ralston, 2010; Ofek *et al*., 2011). The endocannabinoids anandamide (AEA) and 2‐arachidonoylglycerol (2‐AG) and a variety of plant cannabinoids act as an agonist at both Cnr1 and Cnr2 receptors albeit with different degrees of selectivity (Matias *et al*., 2002; Mechoulam, 2005). The endocannabinoids AEA and 2‐AG are produced within the central nervous system and in the bone microenvironment by osteoblasts and osteoclasts (Tam *et al*., 2006, 2008; Rossi *et al*., 2009; Idris & Ralston, 2010; Whyte *et al*., 2011). Agonists of Cnr1 and 2 receptors inhibit adenylyl cyclase causing reduction in intracellular levels of cyclic adenosine monophosphate and activation of a variety of downstream signalling pathways including ion channels, Nuclear Factor Kappa‐B (**NFκB**), phosphoinositide kinase, mitogen‐activated protein kinase and modulation of second messengers such as ceramide and intracellular calcium (Demuth & Molleman, 2006; Idris & Ralston, 2010).

Studies in genetically modified mice implicated both Cnr1 and Cnr2 in the regulation of peak bone mass and age‐related bone loss. We have reported that adult Cnr1 knockout female mice on inbred (ABH) and outbred (CD1) backgrounds have high peak bone mass and are protected against ovariectomy‐induced bone loss due to an osteoclast defect (Idris *et al*., 2005). However, the same animals develop low bone mass with increasing age due to a defect in bone formation (Idris *et al*., 2009). In contrast to these findings, Tam and colleagues reported that female mice with Cnr1 deficiency exhibit low peak bone mass when inbred onto a C57BL/6 background (Tam *et al*., 2006). However, we and others have reported that female Cnr2 knockout mice develop low bone mass with age due to a decrease in bone formation (Ofek *et al*., 2006; Sophocleous *et al*., 2014a,b). Together, these findings demonstrate that both Cnr1 and Cnr2 receptors play a role in bone metabolism but suggest that their effects are to some extent distinct. Studies in humans have shown that heavy use of recreational cannabis, which is known to activate both Cnr1 and 2 receptors, is associated with low bone mineral density and increased bone turnover (Sophocleous *et al*., 2017). As the individual cannabinoid receptors play a role in skeletal homeostasis, we generated mice with combined deficiency in Cnr1 and Cnr2 on the outbred CD1 genetic background and evaluated the consequences of combined deficiency of both Cnr1 and 2 receptors on bone metabolism in mice.

## Results

### Deficiency of Cnr1/2 has no effect on survival or body weight

**Figure 1:**
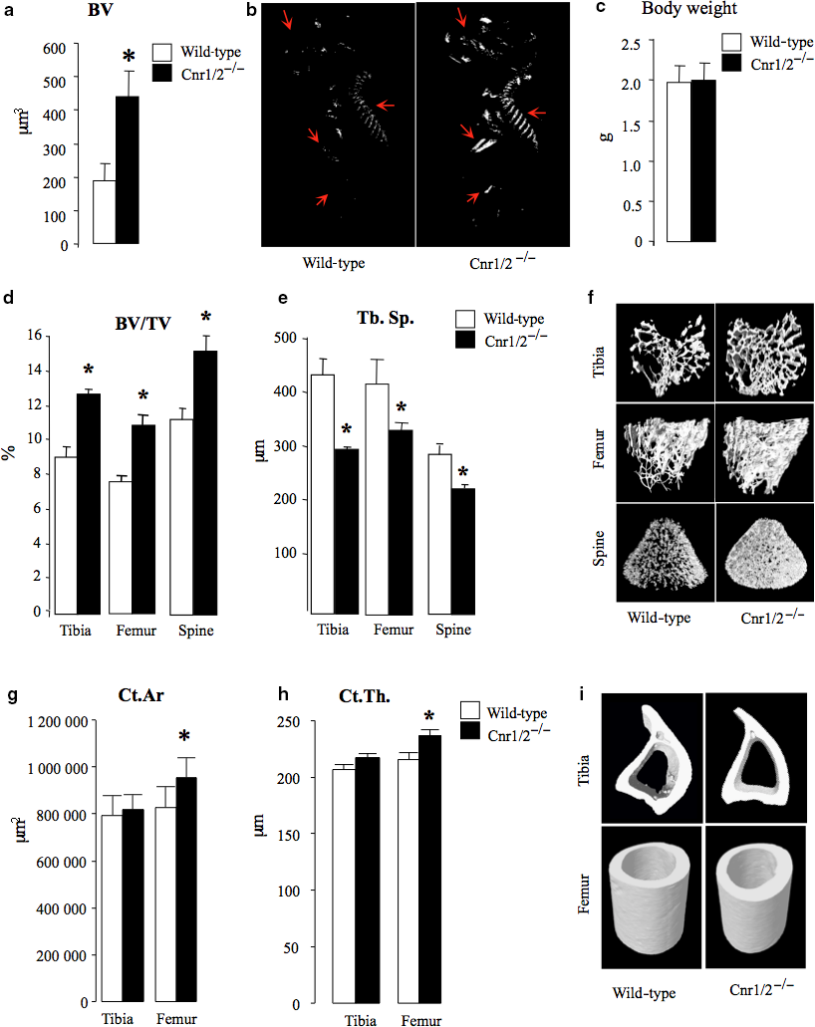
Accelerated bone accrual in neonates and increased trabecular bone mass in adult female mice with combined deficiency in Cnr1 and Cnr2 receptors. (a) Total bone volume (BV, mm^3^) of wild‐type (wt, *n *= 7) and Cnr1/2^−/−^ (*n *= 8) mice at birth as assessed by microCT analysis. (b) Representative microCT images of lateral and posterior view from mice described in panel a. (c) Total body weight (gram) of wild‐type (wt, *n *= 7) and Cnr1/2^−/−^ (*n *= 8) mice from the experiment described in a and b. (d–i) Trabecular and cortical bone parameters in Cnr1/2 double‐knockout (Cnr1/2^−/−^) CD1 mice (*n *= 7) and wild‐type (wt, *n *= 7) control assessed at 3 months of age at the tibial, femur and vertebrae L6 by microCT (d: trabecular volume, BV/TV (%); e, trabecular separation, Tb.Sp. (μm); g, cortical area, Ct.Ar (μm^2^) h; cortical thickness, Ct. Th. (μm). (f and i) Representative microCT images of trabecular (f) and cortical (i) bone from tibiae and femurs of the mice described in panels d, e, g, h. Values are mean ± SEM. *P < 0.05, Cnr1/2^−/−^ versus wild‐type groups.

External examination of neonates and adult (day 1–360) Cnr1/2^−/−^ mice showed no discernible phenotype. The mice exhibited no signs of impaired reproduction and survived normally when compared to wild‐type littermates in the breeding colony. These general observations are similar to those previously reported in mice deficient in the Cnr1 or Cnr2 receptors (Idris *et al*., 2005, 2009; Ofek *et al*., 2006; Sophocleous *et al*., 2014a,b). Body weight is an important determinant of bone mass, and previous studies have shown that both Cnr1 and Cnr2 receptors play a role in the regulation of appetite and body weight (Colombo *et al*., 1998; Kirkham & Williams, 2001; Cota, 2007; Agudo *et al*., 2010). Here, we report that body weight was comparable between Cnr1/2^−/−^ and wild‐type (wt) control mice at birth, 7, 90, 180 and 360 days and after oestrogen cessation in ovariectomized 3‐month‐old mice (Fig. 1c and Table S1).

### Deficiency of Cnr1/2 enhances embryonic bone accrual

Skeletal phenotyping of mice at birth revealed that Cnr1/2^−/−^ female mice had accelerated embryonic bone accrual and exhibited 100% increase in bone volume when compared to wild‐type control mice of similar age (Fig. 1a–b). This suggests combined deficiency of Cnr1 and 2 enhances bone accrual during embryonic development.

### Deficiency of Cnr1/2 increases bone mass during adulthood

We next investigated the effects of combined deficiency of Cnr1 and 2 on bone mass in 3‐month‐old mice. As shown in Fig. 1 (panels d – f), Cnr1/2^−/−^ female mice at this age exhibited a significant increase in trabecular bone volume (Fig. 1d,f) and number (Fig. S2a), coupled with significant reduction in trabecular separation (Fig. 1e) at the tibial and femoral metaphysis and spine. Analysis of cortical bone parameters revealed that Cnr1/2^−/−^ female mice had increased cortical area (Fig. 1g,i) and thickness (Fig. 1h) at the femoral diaphysis and were more resistant to fracture (Fig. 3b). No significant differences between wild‐type and Cnr1/2^−/−^ female mice were detected at the tibial diaphysis at this age. A similar increase in trabecular bone mass (Fig. S2a–b), but not in cortical (Fig. S2c–d), bone mass at the femoral metaphysis was also observed in male Cnr1/2^−/−^ mice of similar age when compared to male wild‐type controls.

### Deficiency of Cnr1/2 attenuates age‐related trabecular bone loss

**Figure 2:**
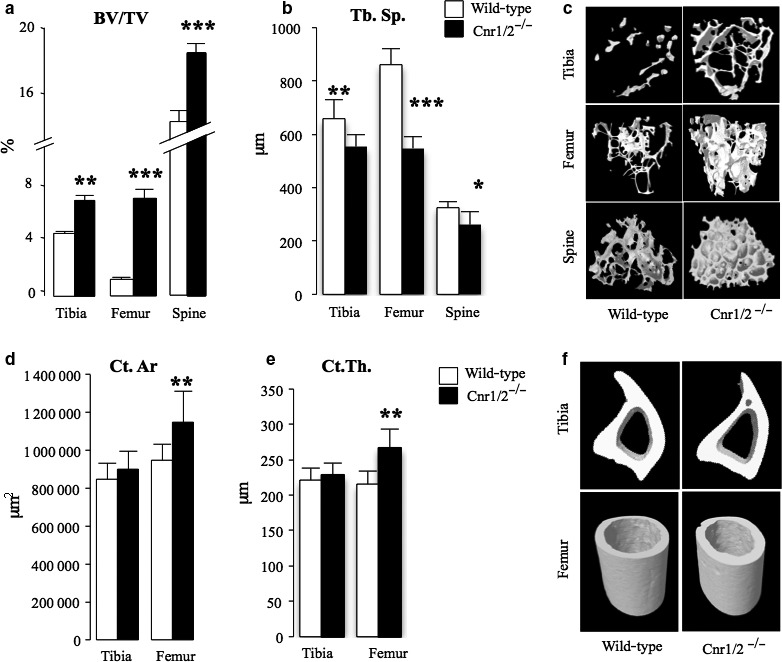
Aged female mice with combined deficiency in Cnr1 and Cnr2 receptors exhibit high bone mass. Trabecular and cortical bone parameters in Cnr1/2 double‐knockout (Cnr1/2^−/−^) CD1 mice (*n *= 9) and wild‐type (wt, *n *= 7) control assessed at 12 months of age at the tibia, femur and vertebrae L6 by microCT (a: trabecular volume, BV/TV (%); b, trabecular separation, Tb.Sp. (μm); d, cortical area, Ct.Ar. (μm^2^) e; cortical thickness, Ct. Th. (μm). (c and f) Representative microCT images of trabecular (c) and cortical bone (f) from tibiae and femur of the mice in panels a, b, d, e. Values are mean ± SEM. Significant differences between WT and Cnr1/2^−/−^ groups are indicated by *P < 0.05, **P < 0.01 and ***P < 0.001.

We went on to examine whether combined deficiency in Cnr1 and Cnr2 affects the development of age‐related bone loss in aged female mice. We observed that both wild‐type and Cnr1/2^−/−^ female mice had lower trabecular bone mass by the age of 12 months as compared with 3 months of age, but trabecular bone mass in Cnr1/2^−/−^ mice at 12 months was better preserved than in wild‐type mice. The values for trabecular bone volume (mean ± SD) were 41.7 ± 11.7 (*n *= 8) and 40.6 ± 14.6% (*n *= 8), lower at the tibial metaphysis and femoral metaphysis, respectively, in Cnr1/2^−/−^ mice compared with 50 ± 4.7% (*n *= 8, *P *< 0.05) and 82 ± 9.7% (*n *= 8, *P* < 0.01) lower in wild‐type controls. MicroCT analysis of the trabecular bone compartment revealed that Cnr1/2^−/−^ mice at this age had significantly high trabecular bone volume and number, and lower trabecular separation at tibial metaphysis, femoral metaphysis and spine (Figs 2a,b and S4a,b). Analysis of cortical bone parameters (Fig. 2d–e) revealed that Cnr1/2^−/−^ exhibited high cortical area and thickness and reduced marrow diameter (Fig. S4d) in the femoral diaphysis, whereas no significant differences between wild‐type and Cnr1/2^−/−^ female mice were detected at the tibial diaphysis (Fig. S4c–d). Representative microCT images of trabecular and cortical bone in Cnr1/2^−/−^ female mice and wild‐type control visualized at this age are shown in Fig. 2, panels c and f. These data indicate that combined deficiency in Cnr1 and 2 protects against age‐related trabecular bone loss in aged female mice.

### Deficiency of Cnr1/2 enhances bone strength in aged mice

**Figure 3:**
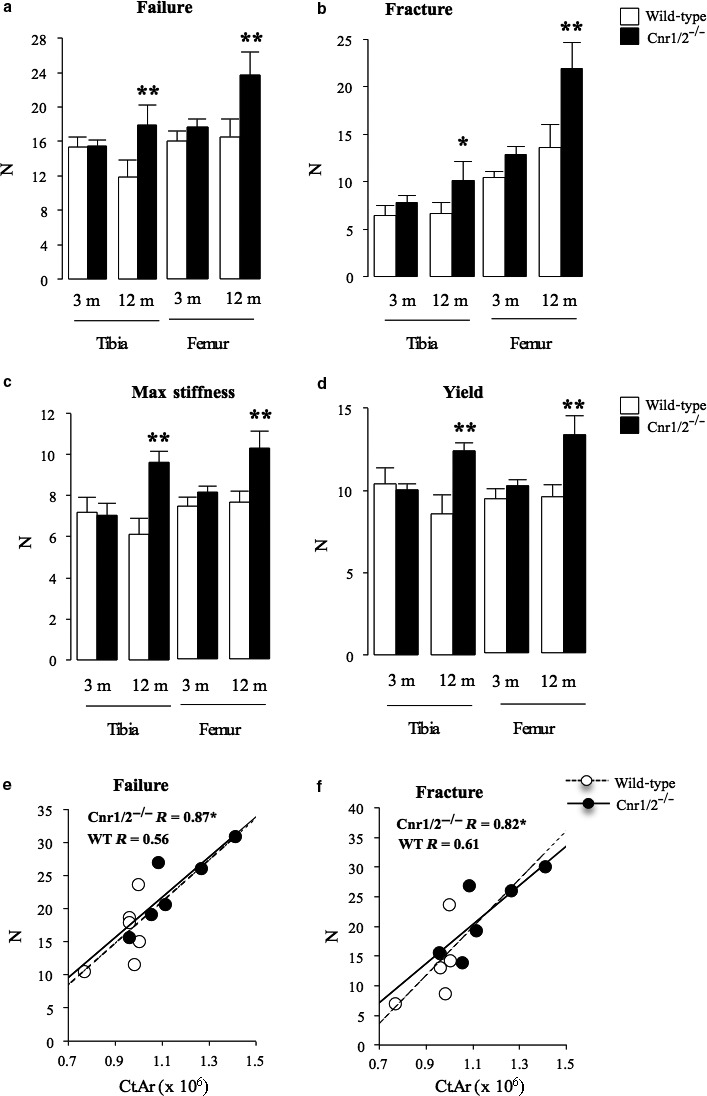
Increased bone strength in aged female mice with combined deficiency in Cnr1 and Cnr2 receptors. Bone strength parameters in Cnr1/2 double‐knockout (Cnr1/2^−/−^) CD1 mice and wild‐type (wt) control assessed at 3 and 12 months of age at the tibial and femoral diaphysis by three‐point bending (a, Failure, Newton; b, Fracture, Newton; c, Maximum stiffness, Newton; d, Yield, Newton). Values are mean ± SEM from at least six mice per group. Significant differences between WT and Cnr1/2^−/−^ groups are indicated by **P < 0.05. Pearson's correlation analyses were performed across 12‐month‐old wild‐type and Cnr1‐ and Cn2‐deficient mice groups between cortical area (Ct.Ar) and peak failure (e) and (f) facture loads at the femoral diaphysis. Pearson correlation coefficients (R) are indicated on each graph. *P < 0.05 indicates significant correlation between variables.

To assess the effect of combined deficiency of Cnr1 and 2 on bone strength, three‐point bending tests were performed in mice of 3 and 12 months of age. As shown in Fig. 3, combined Cnr1 and 2 deficiency was associated with increased tibial and femoral bone strength in 12‐month‐old female mice, with a significant increase (*P* < 0.05) in load to failure (+16% in tibia and +35% in femur) (Fig. 3a); fracture (+29% in tibia and +68% in femur) (Fig. 3b); maximum stiffness (+36% in tibia and +26% in femur) (Fig. 3c); and yield (+24% in tibia and +31% in femur) (Fig. 3d) compared with age‐matched wild‐type controls. Pearson's correlation analysis revealed significant correlations between cortical area and biomechanical variables including stiffness (data not shown), and the peak load for fracture and failure at the femoral diaphysis for the Cnr1‐ and Cnr2‐deficient group at 12 months of age (*P* < 0.05) (Fig. 3e,f). At the tibial diaphysis, only the relationship between cortical area and fracture peak load was significant in Cnr1‐ and Cnr2‐deficient mice (data not shown). The regression lines for the wild‐type group at 12 months of age showed no significant correlation between cortical area and bone strength.

### Effects of oestrogen deficiency and replacement on bone mass in Cnr1/2‐deficient mice

**Figure 4:**
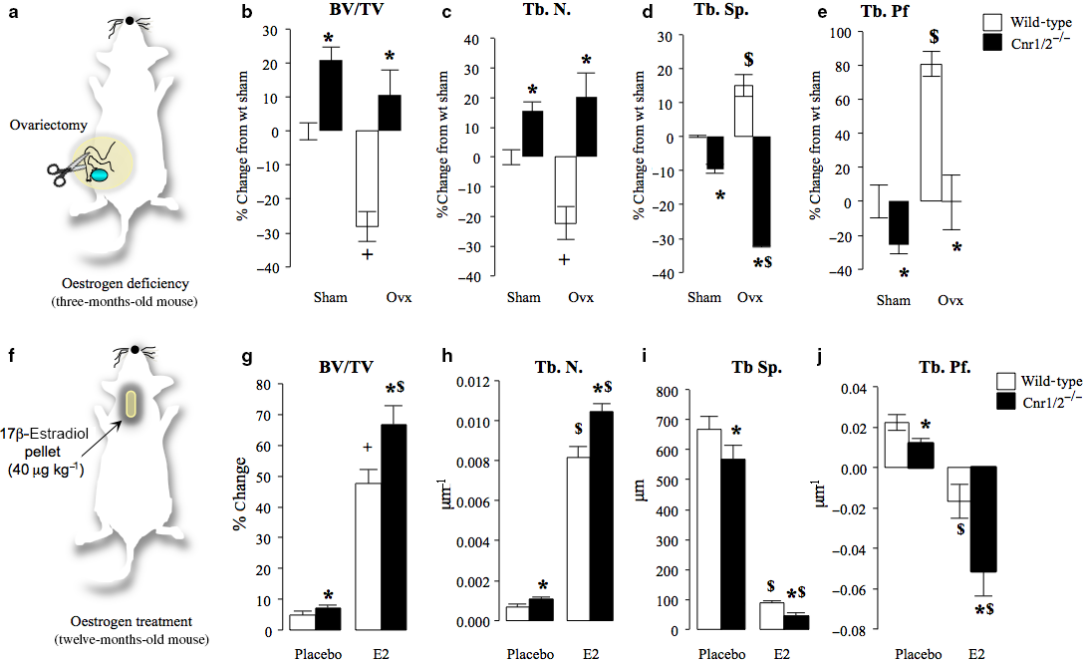
Effects of oestrogen deficiency and replacement on bone mass in Cnr1/2 double‐knockout aged female mice. (a–e) Trabecular bone volume (b, BV/TV, %), number (c, Tb. N., μm^−1^) and separation (d, Tb.Sp., μm) and trabecular pattern factors (e, Tb.Pf, μm^−1^) in sham and ovariectomized 3 month Cnr1/2 double‐knockout (Cnr1/2^−/−^, *n *= 7) CD1 mice and wild‐type control (*n *= 6) assessed at the tibial metaphysis by microCT. (f–j) Trabecular bone parameters in 12‐month‐old Cnr1/2 double‐knockout (Cnr1/2^−/−^) CD1 mice and wild‐type control treated with placebo (*n *= 7) or estradiol (E2, 40 μg kg^−1^ day^−1^, *n *= 7) for 3 months and bone parameters assessed at the tibial metaphysis by microCT (a: bone volume, BV/TV (%); b: trabecular number, Tb. N. (μm^−1^); c: trabecular separation, Tb.Sp. (μm); d, trabecular pattern factor, Tb.Pf. (μm^−1^). Values are mean ± SD. *P < 0.05, wild‐type versus Cnr1/2^−/−^ groups, $*P* < 0.05, wild‐type sham and ovariectomized (ovx) groups and ^+^ *P* < 0.05, placebo and E2 groups.

To determine whether combined deficiency of Cnr1 and Cnr2 modulates the skeletal response to oestrogen, we studied the effects of ovariectomy on bone loss in Cnr1/2^−/−^ and wild‐type female mice at 3 months of age (Fig. 4a). This showed that Cnr1/2^−/−^ mice were partially protected against ovariectomy‐induced bone loss as compared with wild‐type (Fig. 4b–e). Furthermore, the decrease in trabecular bone volume (Fig. 4b) was greater in wild‐type control than in ovariectomized Cnr1/2^−/−^ mice (*P* < 0.05). We also went on to study the effects of oestrogen in older Cnr1/2^−/−^ female mice (Fig. 4f). At 9 months of age, Cnr1/2^−/−^ and wild‐type female mice received either placebo or 17β‐estradiol (E2, 40 μg kg^−1^) pellets. By 12 month of age, E2 significantly increased trabecular bone volume, trabecular number and reduced trabecular separation in both Cnr1/2^−/−^ and wild‐type control mice (Fig. 4, panels g–j). The increase in trabecular bone volume was greater in Cnr1/2^−/−^ mice than in wild‐type mice (*P* < 0.05).

### Bone metabolism in mice with combined deficiency of Cnr1/2 as compared with mice with deficiency of a single receptor

Analysis of the bone parameters reported in this study and those which we previously reported in mice of the same genetic background are shown in Table 2. Mice deficient in Cnr1, Cnr2 or both Cnr1/2 exhibited higher peak bone mass at 3 month of age as compared with wild‐type due to a significant reduction in osteoclast number (Tables 1 and 2). With aging, Cnr1/2^−/−^ continued to have higher bone mass than wild‐type despite a reduction in osteoblast number. This contrasts with the situation in Cnr2^−/−^ mice and Cnr1^−/−^ mice where bone mass was lower in aged mice compared with wild‐type due to an osteoblast defect (Tables 1 and 2). These findings indicate that genetic inactivation of both Cnr1 and Cnr2 receptors affects peak bone mass in a similar way to inactivation of the single receptors. However, with aging, the osteoclast defect in Cnr1/2^−/−^ mice outweighs the effect of the osteoblast defect causing relative preservation of bone mass.

**Table 1:** Static and dynamic histomorphometry in Cnr1/2 double‐knockout (Cnr1/2^−/−^) and wild‐type mice

Age	Genotype	Oc.N/BS (cells mm^−1^)	Act.Res/BS (%)	Ob.N/BS (cells mm^−1^)	MAR (μm day^−1^)	MS/BS (%)	BFR/BS (μ)m^3^ (μ)m^−2^ day^−1^	AdipoS/BS (%)
3 months	Wild‐type	0.78 ± 0.18	2.45 ± 0.19	22.5 ± 1.6	4.1 ± 0.2	51.9 ± 1.5	2.1 ± 0.2	0.013 ± 0.01
	CNR1/2^−/−^	0.60 ± 0.16*	1.9 ± 0.12*	17.9 ± 1.0	4.3 ± 0.1	51.6 ± 0.8	2.2 ± 0.1	0.011 ± 0.01
12 months	Wild‐type	1.7 ± 0.2	4.9 ± 0.5	17.6 ± 1.5	1.7 ± 0.4	46.8 ± 1.7	0.8 ± 0.05	0.09 ± 0.01
	CNR1/2 ^−/−^	1.4 ± 0.1*	4.5 ± 0.4**	11.7 ± 0.1**	1.8 ± 0.1	32.8 ± 2.6**	0.6 ± 0.06**	0.30 ± 0.07**

Act.Res; active resorption surface/bone surface (%); Oc.N/BS, osteoclast number/bone surface (cells/mm); AdipoS/BS, adipocyte surface/bone surface (%); Ob.N/BS, osteoblast number /bone surface (cells/mm); MAR, mineral apposition rate (μm day^−1^); MS/BS, mineralizing surface/bone surface (%); BFR/BS: bone formation rate/bone surface (μ)m^3^ (μ)m^−2^ day^−1^; values are means ± SEM and are obtained from six to eight animals. *P < 0.05, **P < 0.01 between genotype groups.

**Table 2:** Comparison of skeletal phenotype in Cnr1, Cnr2 and Cnr1/2 double‐knockout (Cnr1/2^−/−^) compared to wild‐type mice

Age	Genotype	BV/TV	Ct.Ar	Oc.N/BS	Ob.N/BS	MAR	MS/BS	BFR/BS
3 months	CNR1^−/−^	34. ±7.5	4. ±2.3^ns^	−54.0 ± 4.8*	−7.3 ± 10.9^ns^	10.2 ± 6.6^ns^	−18.4 ± 4.5	−9.1 ± 10.5^ns^
	CNR2^−/−^	55.5 ± 11.2	−1.1 ± 2.1^ns^	−31.4 ± 6.3	4.7 ± 5.6^ns^	−21.3 ± 4.1*	−9.7 ± 3.8^ns^	−26.8 ± 5.4
	CNR1/2^−/−^	39.9 ± 3.4	2.8 ± 2.8^ns^	−22.0 ± 5.2	−20.3 ± 4.6+	5.1 ± 3.5^ns^	−0.7 ± 1.5$ ^ns^	3.4 ± 3.9+ ^ns^
12 months	CNR1^−/−^	−11.3 ± 6.2	−4.0 ± 3.0^ns^	−91.9 ± 4.6*	−54.8 ± 10.7	−55.9 ± 10.5	−6.9 ± 6.7^ns^	−60.4 ± 8.1
	CNR2^−/−^	7.6 ± 7.5^ns^	−4.7 ± 2.3^ns^	0.0 ± 8.6^ns^	−34.4 ± 5.6	−28.3 ± 6.1	−21.5 ± 5.4	−46.3 ± 6.4
	CNR1/2^−/−^	53.3 ± 12.5*	6.4 ± 4.6^ns^	−18.4 ± 6.3^ns^	−33.7 ± 6.2	6.8 ± 5.9* ^ns^	−30.4 ± 5.5	−25.6 ± 7.3

Values are % change from wild‐type. BV/TV; trabecular bone volume; Ct.Ar; cortical area; Oc.N/BS, osteoclast number/bone surface; Ob.N/BS, osteoblast number/bone surface; MAR, mineral apposition rate; MS/BS, mineralizing surface/ bone surface; BFR/BS: bone formation rate/bone surface; ns, not significant from respective wild‐type control. Values are percentage difference from wild‐type and were obtained from six to eight animals (mean ± SEM). *P < 0.05 from all genotype groups, ^+^ *P* < 0.05 from Cnr2^−/−^, ^$^ *P* < 0.05 from Cnr1^−/−^.

### Osteoclast numbers and bone resorption are reduced in Cnr1/2‐deficient mice

**Figure 5:**
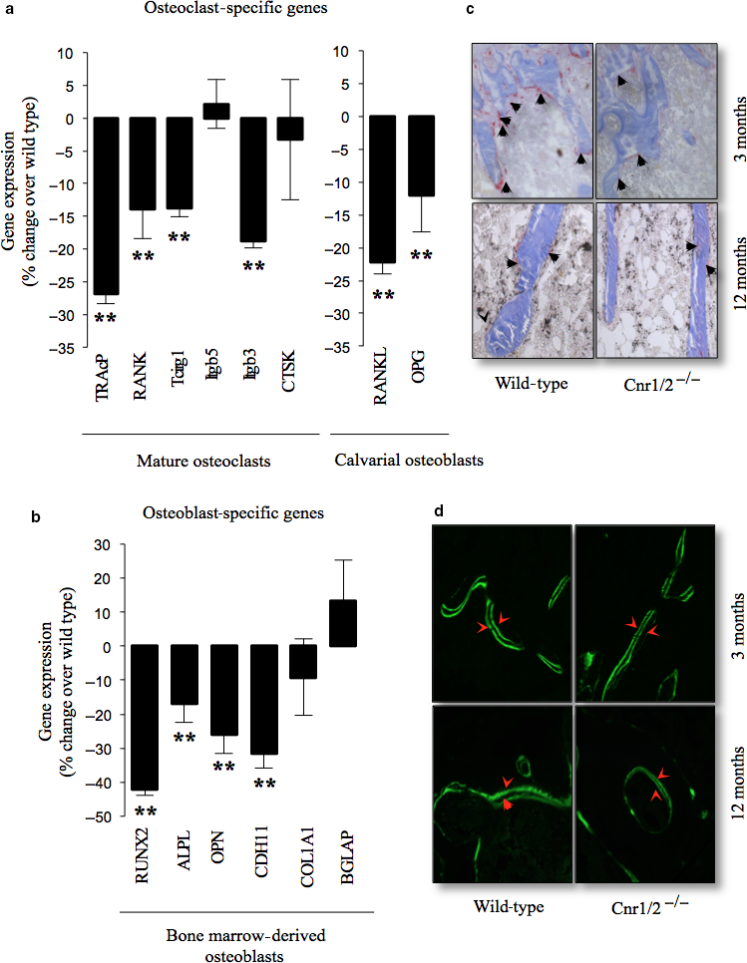
Combined deficiency in Cnr1 and 2 receptors reduces the expression of osteoclast and osteoblast‐specific genes. (a – b) The expression of osteoclast (a)‐ and osteoblast (b)‐specific genes was measured by RT–PCR followed by qPCR. Transcript numbers are expressed as copy number per mg and normalized over of 18s RNA. Values in the graphs are mean* *± SD and are obtained from three independent experiments (*P < 0.05, **P < 0.01, from wild‐type). RANKL‐ and M‐CSF‐generated osteoclast, bone marrow osteoblast and calvarial osteoblast cultures were obtained from three independent experiments. Abbreviations and primers are listed in Table S2. (c – d) Representative photomicrographs of trabecular bone from the tibial metaphysis of Cnr1/2 double‐knockout (Cnr1/2^−/−^) and wild‐type control female mice at 12 months of age showing (c) osteoclasts (tartrate‐resistant acid phosphatase [TRAcP] staining, black arrows) and (d) bone surface (double Calcein labelling, red arrows).

To define the mechanisms responsible for the increased trabecular bone mass associated with Cnr1/2 deficiency, we studied bone histomorphometry in Cnr1/2^−/−^ and wild‐type female mice. This showed that active resorption surface and osteoclast numbers were significantly lower in Cnr1/2^−/−^ when compared with wild‐type at both 3 months and 12 months (Table 1). In keeping with this, quantitative PCR analysis of various osteoclast‐specific genes in mature osteoclast cultures and in calvarial osteoblasts revealed that mRNA levels of acp5 (TRAcP), tnfrsf11a (RANK), Tcirg1 and Itgb3 in osteoclasts (Fig. 5a) and tnfs11 (RANKL) and tnfrsf11b (OPG) in calvarial osteoblasts (Fig. 5a, right panel) were significantly lower in Cnr1/2^−/−^ cultures than in wild‐type control cultures. In contrast, the mRNA expression of Itgb3 and Cathepsin K (CTSK) was not affected in Cnr1/2^−/−^ cultures when compared to cultures from wild‐type controls.

### Bone formation is reduced in aged female mice with Cnr1/2 deficiency

Analysis of bone formation parameters revealed no changes in bone formation and mineral apposition rate at the age of 3 month but mild reduction in osteoblast number was detected in Cnr1/2^−/−^ mice (Table 1). By 12 months of age, Cnr1/2^−/−^ mice suffered a significant reduction in osteoblast number and mineralizing surface (Table 1), but bone mineral apposition rate remained unchanged (Table 1). We also detected a significant reduction in the mRNA expression of the osteoblast‐specific genes Runt Related Transcription Factor 2 (rnx2), alkaline phosphatase (alpl), osteopontin (opn) and cadherin‐11 (cdh11) in bone marrow‐generated osteoblasts obtained from aging Cnr1/2^−/−^ mice at 12 months of age (Fig. 5b–d).

### Blockade of Cnr1, but not Cnr2, enhances adipocyte differentiation

**Figure 6:**
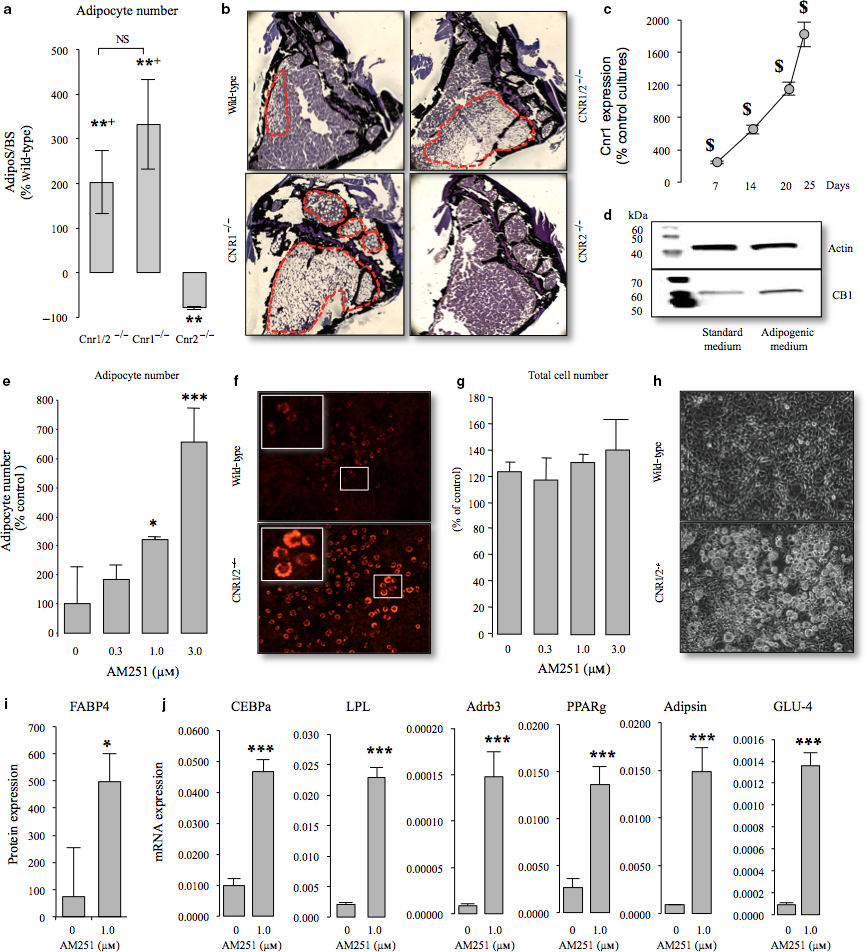
Blockade of Cnr1, but not Cnr2, enhances adipocyte differentiation *in vitro* and *in vivo*. (a) Quantification of adipocyte numbers at the tibial metaphysis in mice with Cnr1, 2 or both receptor deficiencies at 12 months of age. NS, not significantly different from Cnr2; *P < 0.05 from wild‐type; ^+^ *P* < 0.05 from Cnr2. (b) Representative photomicrographs of bone from panel a showing bone (Von Kossa staining, black) and bone marrow adipocyte (dotted line). (c – d) Western blot analysis of the Cnr1 receptor in the preadipocyte 3T3‐L1 cells cultured in standard or adipogenic medium for the indicated period. ^$^ *P* < 0.05 from standard medium treated cultures (e – h) Quantification of adipocyte numbers by staining with Nile Red (e) and viability by AlamarBlue assay (g) in cultures of the preadipocyte 3T3‐L1 cells treated with vehicle (0.1% DMSO) or the CB1‐selective inverse agonist AM251 in the presence of insulin (1 μg mL^−1^) for 10 days. (f and h) Representative photomicrographs of adipocytes in the presence (f) or absence (h) of Nile Red staining (X10 magnification). (i) The protein level of FABP4 in the 3T3‐L1 cells described in panels e – h as assessed by Western blot analysis, (i) The mRNA expression of the adipocyte‐specific genes CCAAT/enhancer binding protein alpha (CEBPα); lipoprotein lipase (LPL); adrenoceptor beta 3 (Adrb3); peroxisome proliferator‐activated receptor gamma (PPARγ); Adipsin (complement factor D, CFD) or beta‐glucosidase 4 (GLU4) in the 3T3‐L1 cells described in panels e – i as assessed by RT–PCR followed by qPCR, (j) in the 3T3‐L1 cells described in panels e – h as assessed by Western blot analysis and qPCR, respectively. *P < 0.05 and ***P < 0.001 from vehicle treated. Transcript numbers are expressed as copy number per mg of 18s RNA. Data are from three independent experiments (*n *= 3). Abbreviations and primers are listed in Table S2.

Histological analysis of the bone marrow compartment revealed increased numbers and accumulation of adipocytes in both Cnr1/2^−/−^ and Cnr1^−/−^ mice, but this was more evident in Cnr1^−/−^ mice where the trabecular marrow space was almost completely replaced with adipocytes (Fig. 6a,b). The difference in adipocyte number that was observed in Cnr1/2^−/−^ knockout was not significantly different to those observed in single knockouts of Cnr1 (Fig. 6a). In contrast, the space occupied by adipocytes in the bone marrow compartment of female Cnr2^−/−^ mice at this age was significantly lower than in Cnr1^−/−^ and Cnr1/2^−/−^ mice (Fig. 6a). Mice with deficiency of the Cnr1 receptor exhibited a significant reduction in bone marrow fat accumulation compared to wild‐type controls (Fig. 6b), confirming the role of Cnr1 in this process. Taken together these observations suggest that Cnr1 plays a more important role than Cnr2 in regulating bone marrow adipocyte differentiation. To further investigate the contribution of Cnr1 to adipocyte differentiation, we studied the expression of Cnr1 and a number of adipocyte‐specific genes in cultures of the preadipocyte 3T3‐L1 cells in the presence and absence of adipogenic medium and the Cnr1‐selective inverse agonist AM251. We observed a significant increase in the protein level of Cnr1 in the cultures of the preadipocyte 3T3‐L1 exposed to adipogenic medium (Fig. 6c,d) and showed that under these conditions, the Cnr1‐selective inverse agonist AM251 increased adipocyte number (Fig. 6e–f), enhanced the protein expression of the adipocyte‐specific FABP4 (Fig. 6i) and significantly increased the mRNA expression of CCAAT/enhancer binding protein alpha (CEBPα), lipoprotein lipase (LPL), adrenoceptor beta 3 (Adrb3), peroxisome proliferator‐activated receptor gamma (PPARγ), Adipsin (CFD, complement factor D) and beta‐glucosidase 4 (GLU4), all of which are key regulators of adipocyte differentiation and maturation (Champigny *et al*., 1992; Ntambi & Young‐Cheul, 2000; Burton *et al*., 2004). It is important to note that AM251 had no significant effects on the proliferation of 3T3‐L1 under these conditions excluding the possibility that the stimulatory effect on adipogenesis was mediated by an increase in cell number (Fig. 6g–h).

## Discussion

Over recent years, there has been increasing interest in the role that endocannabinoids and their receptors play in the regulation of bone metabolism (Bab *et al*., 2009; Idris & Ralston, 2012). Genetic inactivation of Cnr1on an outbred (CD1) background is associated with increased peak bone mass due to reduced osteoclast number but is associated with lower bone mass with age due to reduced bone formation and accumulation of adipocytes in the bone marrow. Mice deficient in Cnr2 on the same background also have higher peak bone mass than wild‐type but developed lower bone mass on aging, due to reduced bone formation. Unlike Cnr1^−/−^ mice, elderly Cnr2^−/−^ mice do not accumulate fat in the bone marrow. The observations presented here show that combined deficiency in Cnr1 and Cnr2 enhances bone accrual and increases peak bone mass in adult mice due to an effect on osteoclast number and function. In addition, we detected a small reduction in osteoblast number in Cnr1/2^−/−^ mice, but bone formation parameters including bone formation rate and mineral apposition rate remained unchanged. The differences in peak trabecular bone mass and osteoclast and osteoblast numbers were quantitatively similar to those previously observed in single knockout of Cnr1 and 2 of a similar age on the same genetic background (Idris *et al*., 2009; Sophocleous *et al*., 2014a,b). Collectively, our results suggest that enhanced bone accrual during skeletal growth and increased levels of trabecular bone mass associated with single or double Cnr1 and 2 deficiencies was most probably driven by a reduction in osteoclast number.

Oestrogen positively regulates peak bone mass, and oestrogen deficiency is associated with increased bone loss (Richelson *et al*., 1984; Pacifici, 1996; Raisz, 1996). Previous studies have shown that single deficiency in Cnr1 or Cnr2 in mice is associated with reduced bone density with age and that pharmacological blockade of these receptors causes osteoclast inhibition and prevents bone loss in ovariectomized adult mice (Idris *et al*., 2005, 2009; Sophocleous *et al*., 2011). Here, we observed that Cnr1/2 double‐knockout mice were also protected against ovariectomy‐induced bone loss in adult mice, with results identical to those observed with the single Cnr1 or Cnr2 knockout mice and after administration of pharmacological blockers of these receptors (Idris *et al*., 2005, 2008, 2009). In addition, Cnr1/2 double‐knockout mice at 12 month of age were relatively protected against age‐related bone loss compared with wild‐type. This was associated with an increase in energy to fracture and failure, suggesting the increased cortical bone mass was preserving bone strength. These data suggest that combined deficiency in Cnr1 and 2 has a positive effect on bone density and strength in aged female mice, which is in contrast with previous studies that have shown that single deficiency of Cnr1 or Cnr2 in female mice is associated with increased aged‐related bone loss (Ofek *et al*., 2006; Idris *et al*., 2009; Sophocleous *et al*., 2014a). It is important to note that female mice with combined deficiency in Cnr1 and Cnr2 used in this study – unlike Cnr1‐deficient mice of similar age (Idris *et al*., 2005, 2009) – showed no significant difference in body weight throughout life when compared to wild‐type control. These findings indicate that combined deficiency in Cnr1 and 2 enhances bone mass by an effect that is independent of body weight.

Histomorphometric analysis in aged Cnr1/2 double‐knockout mice showed a reduction in osteoclast and osteoblast numbers, a reduction in bone formation rate and an increase in bone marrow fat accumulation. The reduction in bone formation is consistent with previous studies in single Cnr1 and 2 mice (Table 2 and (Ofek *et al*., 2006; Tam *et al*., 2008; Ofek *et al*., 2011; Sophocleous *et al*., 2011) and with previous work that showed that cannabinoid receptor activation in osteoblasts exerts an anabolic effect (Ofek *et al*., 2006, 2011; Sophocleous *et al*., 2011). We speculate that the likely mechanism for the higher bone mass in aged Cnr1/2 double‐knockout mice compared with single cannabinoid receptor deficiency is a relative reduction in osteoclast number which outweighs the reduction in osteoblast activity and increased adipocyte differentiation that was also observed.

Previous studies using Cnr1 knockout mice have shown an increase in adipocyte accumulation in the bone marrow that was found to be associated with a decrease in osteoblast differentiation and function (Idris *et al*., 2009). In contrast, our present data and those reported by our group in previous studies have demonstrated that aged Cnr2‐deficient mice in both CD1 and C57BL/6 backgrounds have fewer adipocytes in the bone marrow compartments than wild‐type littermates(Sophocleous *et al*., 2014a). Here, we found that aged Cnr1/2‐deficient mice had a similar degree of adipocyte accumulation to mice with single deficiency of Cnr1 (Idris *et al*., 2009). This demonstrates that the Cnr1 plays a more important role than Cnr2 in regulating adipocyte differentiation in bone marrow. In keeping with this, we observed that Cnr1 expression was significantly enhanced following exposure of the preadipocyte 3T3‐L1 cells to adipogenic medium *in vitro* and activation of Cnr1 by the selective inverse agonist AM251 (Champigny *et al*., 1992; Ntambi & Young‐Cheul, 2000; Burton *et al*., 2004).

In conclusion, our present data together with previous results obtained with single Cnr1 or Cnr2 (Table 2) knockout mice and after administration of the Cnr1 AM251 (Idris *et al*., 2005, 2009) and Cnr2 AM630 (Idris *et al*., 2008)‐selective blockers suggest that Cnr1 and 2 have distinct roles in skeletal homeostasis and their combined inhibition protects against age‐related bone loss primarily by inhibiting bone resorption. These findings indicate that combined inhibition of both Cnr1 and Cnr2 receptors could be of value in the prevention of age‐related bone loss due to their inhibitory effects on osteoclast number, although it remains to be seen whether this strategy would have clinical utility given the many other osteoclast inhibitors that are currently available for clinical use.

## Experimental procedures

### Animal experiments

Animals were housed under standard conditions of temperature (25 ± 1°C) and relative humidity (60 ± 10%) on 12‐hour light/dark cycle with *ad libitum* access to standard pellet diet and tap water.

### Generation of Cnr1 and Cnr2 double‐knockout mice on a CD1 genetic background

Mice with targeted inactivation of Cnr1 on the inbred C57BL/6 background were generated by homologous recombination as described previously (Ledent *et al*., 1999; Marsicano *et al*., 2002) and were a kind gift from Dr Christine Ravinet‐Trillou (Sanofi‐Aventis). Mice with Cnr2 deficiency were obtained from Dr Susana Winfield at the NIH and were generated as previously described (Sophocleous *et al*., 2011). These mice had previously been crossed with wild‐type C57BL/6 mice for at least 10 generations to create a congenic strain on a C57BL/6 background. Heterozygous mice from Cnr1 or Cnr2 on the C57BL/6 were bred for at least 17 generations onto a CD1 background before generating the wild‐type and Cnr1/2^−/−^ from different litters. The Cnr1 and 2 mice used in this study were generated by mating of heterozygote breeding pairs. A congenic strain of mice with combined inactivation of both receptors was generated by crossing a homozygote Cnr1 male and homozygote Cnr2 female on the CD1 background (Fig. S1a). Combined inactivation of Cnr1 and 2 genes in homozygous Cnr1/2^−/−^ mice was confirmed by absence of Cnr1 and 2 cDNA (Fig. S1b). Body weight was monitored at the same time at day 1 and 7 and after 3, 6 and 12 months of age.

### Ovariectomy and hormone replacement

All animal experiments were approved by the Animal Welfare and Ethical Review Body of the University of Edinburgh and conducted in accordance with the UK Animals (Scientific Procedures) Act 1986. Ovariectomy or sham ovariectomy was performed in 3‐month‐old adult female mice as previously described (Idris, 2012). The experiment was terminated on day 14, and bone mineral density was measured at the tibial metaphysis by microcomputed tomography (microCT). 17β‐Estradiol (E2, 40 μg kg^−1^ day^−1^) or placebo treatments were administered by slow‐release pellets (Innovative Research of America, Sarasota, Florida, USA) implanted subcutaneously as previously described (Armour *et al*., 2001).

### Microcomputed tomography

Trabecular and cortical bone microCT analysis was performed as previously describe in (Campbell & Sophocleous, 2014), using a Skyscan 1172 instrument (Brucker, Belgium) set at 60 kV and 150 μA and at a resolution of 5 μm. Images were then reconstructed by the Skyscan NRecon program and analysed using Skyscan CTAn software. The whole embryos or adult (3‐month‐old) and aging (12‐month‐old) mice were analysed as previously described (Campbell & Sophocleous, 2014). Trabecular bone was analysed at the left proximal tibial (200 slices directly distal of the growth plate) and distal femoral metaphysis (200 slices directly proximal of the growth plate) and at the sixth lumbar vertebra (L6), the ROI encompassed the secondary spongiosa of the vertebral body extending 1 mm caudally from the cranial spongiosa. Analysis of cortical bone focused on a region extending 1.25 mm distally from the mid‐point of the femoral diaphysis and a region extending 0.5 mm distally from the mid‐point of the tibia.

### Bone histomorphometry

Bone histomorphometry was performed on the proximal tibial metaphysis from wild‐type and Cnr1/2^−/−^ mice. Intraperitoneal injections of 200 μL of Calcein (20 mg kg^−1^, Sigma‐Aldrich, Dorset, UK) were given on day 6 and day 2 prior to the mice being sacrificed in order to assess dynamic histomorphometry. The bones were fixed, processed for static and dynamic histomorphometry according to standard techniques and sectioned using a Leica microtome (Solms, Germany). Sections were stained with von Kossa, Paragon, tartrate‐resistant acid phosphatase (TRAcP) and aniline blue as previously described (Erben & Glosmann, 2012). Static and dynamic bone histomorphometry were performed on trabecular bone focusing on an area between 0.1 and 1.0 mm distal to the growth plate, using custom‐built software‐generated in‐house based on the Aphelion Image Analysis tool kit (Adcis, He′rouville‐Saint‐Clair, France). Three sections, 20 μm apart, were analysed per sample; and each section was analysed in up to five microscopic fields of view.

### Biomechanical Testing

Biomechanical testing was performed on tibiae and femorae that had been kept in sterile PBS and was carried out as previously described (Sophocleous *et al*., 2014a). Briefly, long bones were placed in a Zwick/Roell materials testing machine (Zwick Testing Machines, Leominster, UK) and subjected to three‐point bending at the diaphysis using a 50‐N Zwick/Roell load cell. The span was fixed at 7 mm, and the moving cross head was lowered at 0.4 mm min^−1^. All samples were tested to fracture. Failure and fracture points, energy spent to reach these points and maximum stiffness and yield points were identified from the load–extension curve. Yield was calculated as the point at which a regression line that represents a 5% loss in stiffness crosses the load–displacement curve as described in (Jepsen *et al*., 2015). A sigmoidal curve fitted to the rising portion of the load–extension curve was prepared using GraphPad Prism 4 for Windows (GraphPad Software, San Diego, CA, USA).

### Cultures and reagents

All chemicals and reagents were purchased from Sigma‐Aldrich unless otherwise stated. The Cnr1‐selective inverse agonist AM251 was purchased from Tocris Biosciences (Bristol, UK). The mouse preadipocyte 3T3‐L1 cells were purchased from Sigma‐Aldrich (Ntambi & Young‐Cheul, 2000) and were cultured in standard (D‐MEM supplemented with 10% foetal calf serum (FCS), 5% L‐glutamine, 100 U mL^−1^ penicillin and 100 μg mL^−1^ streptomycin) or adipogenic medium (standard medium supplemented with insulin (1 μg mL^−1^), indomethacin (0.1 μm) and dexamethasone (250 nm). Mouse osteoclasts were generated from bone marrow (BM) cells as previously described in Sophocleous *et al*. (2015). Primary BM osteoblasts were generated from bone marrow (BM) cells in standard alpha‐MEM, supplemented with 10% FCS, penicillin and streptomycin, β‐glycerol phosphate (10 μm) and L‐ascorbic acid (50 μg mL^−1^) (Idris *et al*., 2009). Primary calvarial osteoblasts were isolated from the calvarial bones of two‐day‐old mice by sequential collagenase digestion as previously described (Taylor *et al*., 2014).

### Measurement of protein expression

Western blot analysis was used to detect Cnr1 and FABP4 expression in cultured mouse preadipocyte 3T3‐L1 cells as previously described in Sophocleous *et al*. (2015). Protein concentration was determined using BCA assay (Pierce, USA). Total protein (70 μg) was resolved by SDS–PAGE, transferred onto PVDF membranes (Bio‐RAD, Hamburg, Germany) and immunoblotted with antibodies according to manufacturer's instructions. Detection of human FABP4 (Cell Signalling Technology, Danvers, Massachusetts, USA) and mouse Cnr1 (Cayman Chemical, Hamburg, Germany) was performed using rabbit monoclonal antibodies (Cell Signalling Technology) followed by appropriate secondary antibody coupled to horseradish peroxidase and then visualized using chemiluminescence (Amersham, Hamburg, Germany) on a Syngene GeneGnome imaging system. The intensity of the bands was quantified using GeneSnap software (Syngene, UK), and level of actin expression was used for normalization.

### Quantification of adipocyte function and viability

Nile Red staining was used to assess adipocyte function by visualizing lipid droplets in 3T3‐L1 cells as described in Greenspan *et al*. (1985). AlamarBlue assay was used to assess cell viability as described in Gloeckner *et al*. (2001).

### Assessment of gene expression

Gene expression was detected using quantitative PCR (qPCR) as previously described (Aitken *et al*., 2009). Briefly, cells were lysed using TRIzol reagent, separated with chloroform and precipitated with isopropyl alcohol. The pellet was suspended in DEPC‐treated water, and samples were quantified using a Nanodrop ND‐8000. Complementary DNA (cDNA) was generated using Invitrogen SuperScript III Reverse Transcriptase kit according to manufacturer's instructions. Quantitative real‐time PCR (qPCR) was performed on the cDNA using mouse targeted primer/probe combination sets designed according to the Roche Universal Probe Library (UPL). For verification of amplification, the product was cleaned using a PCR purification kit (Invitrogen) and then run and visualized on a 1.5% agarose gel adjacent to a low‐molecular‐weight ladder. Full list of primers used for amplification of osteoclast, osteoblast and adipocyte‐specific genes is shown in Table S2. Levels of gene expression were expressed as copy number per microgram of total RNA, and 18s was used for cDNA normalization.

### Statistical analysis

Statistical analyses were performed using IBM (Armonk, NY, USA) SPSS Statistics, version 19. Comparison between groups was made by independent‐samples t‐test, and the significance level was set at *P* = 0.05. Pearson's correlation coefficients (R) values were calculated by bivariate correlation with a two‐tailed test of significance. Significant differences between genotypes with regard to % change from their own wild‐type were assessed using one‐way analysis of variance (ANOVA) followed by Tukey HSD post hoc test (for equal variances) or Games–Howell post hoc test (for unequal variances). The significance level was set at *P* = 0.05

## Funding info

This research was funded by a programme grant from Arthritis Research UK (reference 17713).

## Author contributions

Antonia Sophocleous involved in experimental work and data analysis; Silvia Marino involved in experimental work and data analysis; Dilruba Kabir involved in experimental work; Stuart H. Ralston was principal investigator and involved in conception, editing and writing; and Aymen I. Idris involved in conception, experimental, analysis, editing and writing.

## Conflict of interest

A. Sophocleous, S. Marino and D. Kabir report that they have no conflict of interest.

## Supporting information


**Fig. S1** Method of generation of Cnr1/2 double knockout mice.
**Fig. S2** Increased trabecular bone mass in adult male mice with combined deficiency in Cnr1 and Cnr2 receptors.
**Fig. S3** Increased trabecular bone mass in adult female mice with combined deficiency in Cnr1 and Cnr2 receptors.
**Fig. S4** Increased bone mass in aged female mice with combined deficiency in Cnr1 and Cnr2 receptors.
**Table S1** Body weight in Cnr1/2 double knockout and wild type control mice
**Table S2** Primers used for amplification of mouse and human osteoclast, osteoblast and adipocyte specific genes.

## References

[acel12638-bib-0001] Agudo J , Martin M , Roca C , Molas M , Bura AS , Zimmer A , Bosch F , Maldonado R . (2010) Deficiency of CB2 cannabinoid receptor in mice improves insulin sensitivity but increases food intake and obesity with age. Diabetologia 53, 2629–2640. 20835701 10.1007/s00125-010-1894-6

[acel12638-bib-0002] Aitken SJ , Landao‐Bassonga E , Ralston SH , Idris AI (2009) Beta2‐adrenoreceptor ligands regulate osteoclast differentiation in vitro by direct and indirect mechanisms. Arch. Biochem. Biophys. 482, 96–103. 19059194 10.1016/j.abb.2008.11.012

[acel12638-bib-0003] Armour KE , Armour KJ , Gallagher ME , Godecke A , Helfrich MH , Reid DM , Ralston SH (2001) Defective bone formation and anabolic response to exogenous estrogen in mice with targeted disruption of endothelial nitric oxide synthase. Endocrinology 142, 760–766. 11159848 10.1210/endo.142.2.7977

[acel12638-bib-0004] Bab I , Zimmer A , Melamed E (2009) Cannabinoids and the skeleton: From marijuana to reversal of bone loss. Ann. Med., 41, 560–7. 19634029 10.1080/07853890903121025

[acel12638-bib-0005] Burton GR , Nagarajan R , Peterson CA , McGehee RE Jr (2004) Microarray analysis of differentiation‐specific gene expression during 3T3‐L1 adipogenesis. Gene 329, 167–185. 15033539 10.1016/j.gene.2003.12.012

[acel12638-bib-0006] Campbell GM , Sophocleous A (2014) Quantitative analysis of bone and soft tissue by micro‐computed tomography: applications to ex vivo and in vivo studies. BoneKEy Rep. 3, 564. 25184037 10.1038/bonekey.2014.59 PMC4140449

[acel12638-bib-0007] Champigny O , Holloway BR , Ricquier D (1992) Regulation of UCP gene expression in brown adipocytes differentiated in primary culture. Effects of a new beta‐adrenoceptor agonist. Mol. Cell. Endocrinol. 86, 73–82. 1355051 10.1016/0303-7207(92)90177-8

[acel12638-bib-0008] Colombo G , Agabio R , Diaz G , Lobina C , Reali R , Gessa GL (1998) Appetite suppression and weight loss after the cannabinoid antagonist SR 141716. Life Sci. 63, PL113–PL117. 9718088 10.1016/s0024-3205(98)00322-1

[acel12638-bib-0009] Cota D (2007) CB1 receptors: emerging evidence for central and peripheral mechanisms that regulate energy balance, metabolism, and cardiovascular health. Diabetes Metab Res Rev. 23, 507–517. 17683024 10.1002/dmrr.764

[acel12638-bib-0010] Demuth DG , Molleman A (2006) Cannabinoid signalling. Life Sci. 78, 549–563. 16109430 10.1016/j.lfs.2005.05.055

[acel12638-bib-0011] Erben RG , Glosmann M (2012) Histomorphometry in rodents. Methods Mol. Biol. 816, 279–303. 22130936 10.1007/978-1-61779-415-5_19

[acel12638-bib-0012] Gloeckner H , Jonuleit T , Lemke HD (2001) Monitoring of cell viability and cell growth in a hollow‐fiber bioreactor by use of the dye Alamar Blue. J. Immunol. Methods 252, 131–138. 11334972 10.1016/s0022-1759(01)00347-7

[acel12638-bib-0013] Greenspan P , Mayer EP , Fowler SD (1985) Nile red: a selective fluorescent stain for intracellular lipid droplets. J. Cell Biol. 100, 965–973. 3972906 10.1083/jcb.100.3.965 PMC2113505

[acel12638-bib-0014] Idris AI (2012) Ovariectomy/orchidectomy in rodents. Methods Mol. Biol. 816, 545–551. 22130951 10.1007/978-1-61779-415-5_34

[acel12638-bib-0015] Idris AI , Ralston SH (2010) Cannabinoids and bone: friend or foe? Calcif. Tissue Int. 87, 285–297. 20532878 10.1007/s00223-010-9378-8

[acel12638-bib-0016] Idris AI , Ralston SH (2012) Role of cannabinoids in the regulation of bone remodeling. Front Endocrinol.(Lausanne). 3, 136. 23181053 10.3389/fendo.2012.00136 PMC3499879

[acel12638-bib-0017] Idris AI , Van‘t Hof RJ , Greig IR , Ridge SA , Baker D , Ross RA , Ralston SH (2005) Regulation of bone mass, bone loss and osteoclast activity by cannabinoid receptors. Nat. Med. 11, 774–779. 15908955 10.1038/nm1255 PMC1430341

[acel12638-bib-0018] Idris AI , Sophocleous A , Landao‐Bassonga E , van't HR , Ralston SH (2008) Regulation of bone mass, osteoclast function, and ovariectomy‐induced bone loss by the type 2 cannabinoid receptor. Endocrinology 149, 5619–5626. 18635663 10.1210/en.2008-0150

[acel12638-bib-0019] Idris AI , Sophocleous A , Landao‐Bassonga E , Canals M , Milligan G , Baker D , van't HR , Ralston SH (2009) Cannabinoid receptor type 1 protects against age‐related osteoporosis by regulating osteoblast and adipocyte differentiation in marrow stromal cells. Cell Metab. 10, 139–147. 19656492 10.1016/j.cmet.2009.07.006

[acel12638-bib-0020] Jepsen KJ , Silva MJ , Vashishth D , Guo XE , van der Meulen MC (2015) Establishing biomechanical mechanisms in mouse models: practical guidelines for systematically evaluating phenotypic changes in the diaphyses of long bones. J. Bone Miner. Res. 30, 951–966. 25917136 10.1002/jbmr.2539 PMC4794979

[acel12638-bib-0021] Kirkham TC , Williams CM (2001) Endogenous cannabinoids and appetite. Nutr. Res. Rev. 14, 65–86. 19087417 10.1079/NRR200118

[acel12638-bib-0022] Ledent C , Valverde O , Cossu G , Petitet F , Aubert JF , Beslot F , Bohme GA , Imperato A , Pedrazzini T , Roques BP , Vassart G , Fratta W , Parmentier M (1999) Unresponsiveness to cannabinoids and reduced addictive effects of opiates in CB1 receptor knockout mice. Science 283, 401–404. 9888857 10.1126/science.283.5400.401

[acel12638-bib-0023] Marsicano G , Wotjak CT , Azad SC , Bisogno T , Rammes G , Cascio MG , Hermann H , Tang J , Hofmann C , Zieglgansberger W , Di Marzo V , Lutz B (2002) The endogenous cannabinoid system controls extinction of aversive memories. Nature 418, 530–534. 12152079 10.1038/nature00839

[acel12638-bib-0024] Matias I , Pochard P , Orlando P , Salzet M , Pestel J , Di MV (2002) Presence and regulation of the endocannabinoid system in human dendritic cells. Eur. J. Biochem. 269, 3771–3778. 12153574 10.1046/j.1432-1033.2002.03078.x

[acel12638-bib-0025] Matsuda LA , Lolait SJ , Brownstein MJ , Young AC , Bonner TI (1990) Structure of a cannabinoid receptor and functional expression of the cloned cDNA. Nature 346, 561–564. 2165569 10.1038/346561a0

[acel12638-bib-0026] Mechoulam R (2005) Plant cannabinoids: a neglected pharmacological treasure trove. Br. J. Pharmacol. 146, 913–915. 16205721 10.1038/sj.bjp.0706415 PMC1751232

[acel12638-bib-0027] Munro S , Thomas KL , Abu‐Shaar M (1993) Molecular characterization of a peripheral receptor for cannabinoids. Nature 365, 61–65. 7689702 10.1038/365061a0

[acel12638-bib-0028] Ntambi JM , Young‐Cheul K (2000) Adipocyte differentiation and gene expression. J. Nutr. 130, 3122S–3126S. 11110885 10.1093/jn/130.12.3122S

[acel12638-bib-0029] Ofek O , Karsak M , Leclerc N , Fogel M , Frenkel B , Wright K , Tam J , Attar‐Namdar M , Kram V , Shohami E , Mechoulam R , Zimmer A , Bab I (2006) Peripheral cannabinoid receptor, CB2, regulates bone mass. Proc. Natl Acad. Sci. USA 103, 696–701. 16407142 10.1073/pnas.0504187103 PMC1334629

[acel12638-bib-0030] Ofek O , Attar‐Namdar M , Kram V , Dvir‐Ginzberg M , Mechoulam R , Zimmer A , Frenkel B , Shohami E , Bab I (2011) CB2 cannabinoid receptor targets mitogenic Gi protein‐cyclin D1 axis in osteoblasts. J. Bone Miner. Res. 26, 308–316. 20803555 10.1002/jbmr.228 PMC3179350

[acel12638-bib-0031] Pacifici R (1996) Estrogen, cytokines, and pathogenesis of postmenopausal osteoporosis. J. Bone Miner. Res. 11, 1043–1051. 8854239 10.1002/jbmr.5650110802

[acel12638-bib-0032] Pertwee RG , Ross RA (2002) Cannabinoid receptors and their ligands. Prostaglandins Leukot. Essent. Fatty Acids 66, 101–121. 12052030 10.1054/plef.2001.0341

[acel12638-bib-0033] Raisz LG (1996) Estrogen and bone: new pieces to the puzzle. Nat. Med. 2, 1077–1078. 8837601 10.1038/nm1096-1077

[acel12638-bib-0034] Richelson LS , Wahner HW , Melton LJ 3rd , Riggs BL (1984) Relative contributions of aging and estrogen deficiency to postmenopausal bone loss. N. Engl. J. Med. 311, 1273–1275. 6493283 10.1056/NEJM198411153112002

[acel12638-bib-0035] Rossi F , Siniscalco D , Luongo L , De Petrocellis L , Bellini G , Petrosino S , Torella M , Santoro C , Nobili B , Perrotta S , Di MV , Maione S (2009) The endovanilloid/endocannabinoid system in human osteoclasts: possible involvement in bone formation and resorption. Bone 44, 476–484. 19059369 10.1016/j.bone.2008.10.056

[acel12638-bib-0036] Sophocleous A , Landao‐Bassonga E , Van't Hof RJ , Idris AI , Ralston SH (2011) The type 2 cannabinoid receptor regulates bone mass and ovariectomy‐induced bone loss by affecting osteoblast differentiation and bone formation. Endocrinology 152, 2141–2149. 21447627 10.1210/en.2010-0930

[acel12638-bib-0037] Sophocleous A , Idris AI , Ralston SH (2014a) Genetic background modifies the effects of type 2 cannabinoid receptor deficiency on bone mass and bone turnover. Calcif. Tissue Int. 94, 259–268. 24036631 10.1007/s00223-013-9793-8

[acel12638-bib-0038] Sophocleous A , Sims AH , Idris AI , Ralston SH (2014b) Modulation of strain‐specific differences in gene expression by cannabinoid type 2 receptor deficiency. Calcif. Tissue Int. 94, 423–432. 24370613 10.1007/s00223-013-9823-6

[acel12638-bib-0039] Sophocleous A , Marino S , Logan JG , Mollat P , Ralston SH , Idris AI (2015) Bone cell‐autonomous contribution of type 2 cannabinoid receptor to breast cancer induced osteolysis. J. Biol. Chem. 290, 22049–22060. 26195631 10.1074/jbc.M115.649608 PMC4571957

[acel12638-bib-0040] Sophocleous A , Robertson R , Ferreira NB , McKenzie J , Fraser WD , Ralston SH (2017) Heavy cannabis use is associated with low bone mineral density and an increased risk of fractures. Am. J. Med. 130, 214–221. 27593602 10.1016/j.amjmed.2016.07.034

[acel12638-bib-0041] Tam J , Ofek O , Fride E , Ledent C , Gabet Y , Muller R , Zimmer A , Mackie K , Mechoulam R , Shohami E , Bab I (2006) Involvement of neuronal cannabinoid receptor, CB1, in regulation of bone mass and bone remodeling. Mol. Pharmacol. 70, 786–792. 16772520 10.1124/mol.106.026435

[acel12638-bib-0042] Tam J , Trembovler V , Di MV , Petrosino S , Leo G , Alexandrovich A , Regev E , Casap N , Shteyer A , Ledent C , Karsak M , Zimmer A , Mechoulam R , Yirmiya R , Shohami E , Bab I (2008) The cannabinoid CB1 receptor regulates bone formation by modulating adrenergic signaling. FASEB J. 22, 285–294. 17704191 10.1096/fj.06-7957com

[acel12638-bib-0043] Taylor SEB , Shah M , Orriss IR (2014) Generation of rodent and human osteoblasts. BoneKEy Rep. 3, 585. 25396049 10.1038/bonekey.2014.80 PMC4230189

[acel12638-bib-0044] Whyte LS , Ford L , Ridge SA , Cameron GA , Rogers MJ , Ross RA (2011) Cannabinoids and bone: Endocannabinoids modulate human osteoclast function in vitro. Br. J. Pharmacol. 165, 2584–97. 10.1111/j.1476-5381.2011.01519.x PMC3423262 21649637

