# Supplementary material for: Combined deficiency of the Cnr1 and Cnr2 receptors protects against age‐related bone loss by osteoclast inhibition
Source: Aging Cell. 2017 Jul 28;16(5):1051–61. doi: 10.1111/acel.12638 (PMC5595693; doi:10.1111/acel.12638)
Supplement: Supplementary file 1 — Fig. S1 Method of generation of Cnr1/2 double knockout mice. Fig. S2 Increased trabecular bone mass in adult male mice with combined deficiency in Cnr1 and Cnr2 receptors. Fig. S3 Increased trabecular bone mass in adult female mice with combined deficiency in Cnr1 and Cnr2 receptors. Fig. S4 Increased bone mass in aged female mice with combined deficiency in Cnr1 and Cnr2 receptors. Table S1 Body weight in Cnr1/2 double knockout and wild type control mice Table S2 Primers used for amplification of mouse and human osteoclast, osteoblast and adipocyte specific genes. [file ACEL-16-1051-s001.docx]

**Supplementary figures and tables**

**1. Supplementary figures**

**
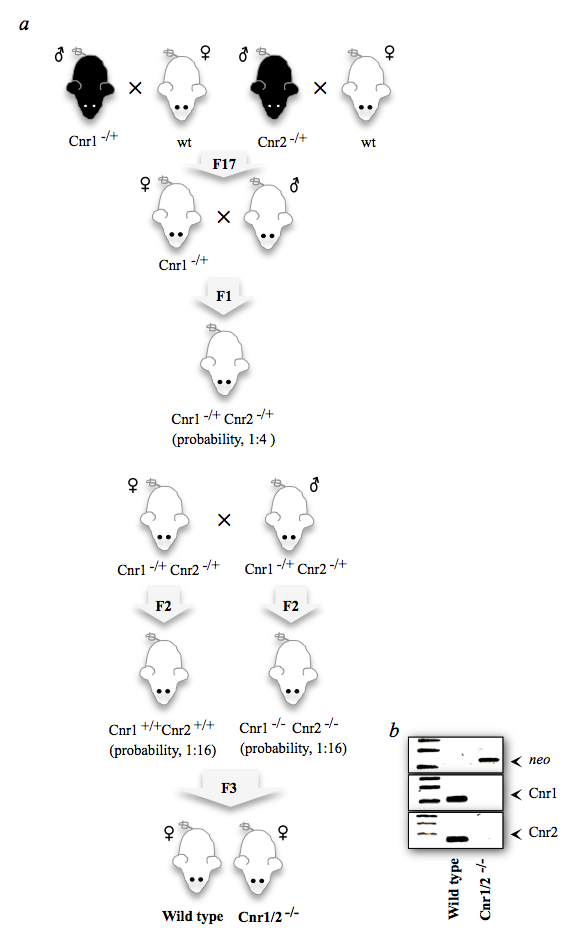
**

**Figure S1. Method of generation of Cnr1/2 double knockout mice.** (a) Graphic representation of generation of Cnr1/2 double knockout mice (Cnr1/2^-/-^) in the outbred CD1 mouse background showing that heterozygous mice from Cnr1 or Cnr2 on the C57BL/6 were bred for at least 17 generations onto a CD1 background before generating the wild type and Cnr1/2^-/-^ from different litters. (b) Neo, Cnr1 and Cnr2 DNA expression in tissue from wild type and Cnr1/2^-/-^ in the outbred CD1 mouse background.

**
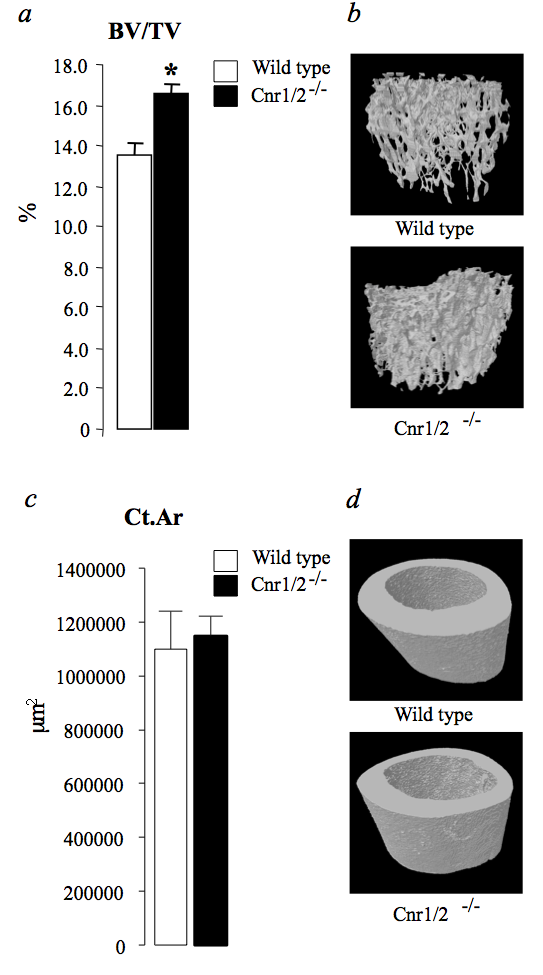
**

**Figure S2. Increased trabecular bone mass in adult male mice with combined deficiency in Cnr1 and Cnr2 receptors.** (a) Trabecular bone volume (BV/TV) in wild type (wt, n = 7) and Cnr1/2^-/-^ (n = 7) mice was assessed at 3 months of age at the femoral metaphysis by microCT. (b) Representative microCT images of trabecular bone from femurs of the mice described in panel a. (c) Cortical area (Ct. Ar) in the mice from experiment described above assessed at the femoral diaphysis by microCT. (d) Representative microCT images of cortical bone from femurs of the mice described in panel c. Values are mean ± SD. Significant differences between wild type and Cnr1/2^-/-^ groups are indicated by *p<0.05.

**
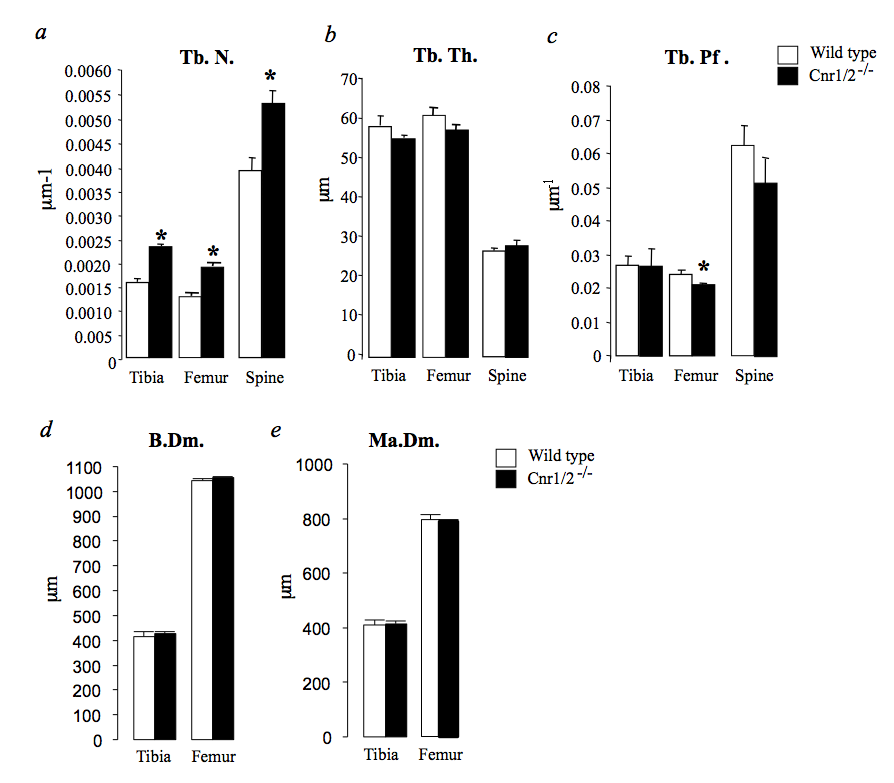
**

**Figure S3. Increased trabecular bone mass in adult female mice with combined deficiency in Cnr1 and Cnr2 receptors.** (a - c) Trabecular bone parameters in wild type (wt, n = 7) and Cnr1/2^-/-^ (n = 7) mice assessed at 3 months of age at the tibial metaphysis, femoral metaphysis and vertebrae L6 by microCT (a: trabecular number, Tb. N. (μm^-1^); b, trabecular thickness, Tb. Th. (μm); c, trabecular pattern factor, Tb.Pf. (μm^-1^). (d – e) Cortical bone parameters in the mice from experiment described above assessed the tibial and femoral diaphysis by microCT (d: bone diameter, B.Dm (μm); e: marrow diameter, Ma.Dm (μm). Values are mean ± SD. Significant differences between wild type and Cnr1/2^-/-^ groups are indicated by *p<0.05.

**
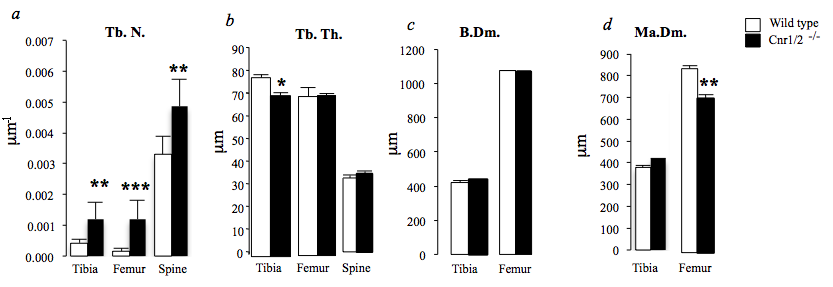
**

**Figure S4. Increased bone mass in aged female mice with combined deficiency in Cnr1 and Cnr2 receptors.** (a - b) Trabecular bone parameters in Cnr1/2 double knockout (Cnr1/2^-/-^) CD1 mice (n = 9) and wild type (wt, n = 7) control assessed at 12 months of age at the tibial metaphysis, femoral metaphysis and vertebrae L6 by microCT (a: trabecular number, Tb. N. (μm^-1^); b, trabecular thickness, Tb. Th. (μm). (c – d) Cortical bone parameters in the mice from experiment described above assessed the tibial and femoral diaphysis by microCT (d: cortical bone diameter, Ct.Dm (μm); e: marrow diameter, Ma.Dm (μm). Values are mean ± SD. Significant differences between wild type and Cnr1/2^-/-^ groups are indicated by *p<0.05.

**2. Supplementary Tables**

**Table S1. Body weight in Cnr1/2 double knockout and wild type control mice**

|  | **Age (days)** | **Phenotype** | |
| --- | --- | --- | --- |
|  |  | wt | Cnr1/2^-/-^ |
|  | 1 | 1.87 ± 0.07 | 2.0 ± 0.19 |
|  | 7 | 3.98 ± 0.19 | 3.76 ± 0.35 |
|  | 90 | 28.8 ± 2.59 | 27.8 ± 3.05 |
|  | 90 (sham) | 27.2 ± 1.66 | 26.9 ± 3.26 |
|  | 90 (Ovx) | 29.1 ± 1.40 | 28.9 ± 2.15 |
|  | 180 | 30.9 ± 1.10 | 32.2 ± 1.77 |
|  | 360 | 33.4 ± 1.50 | 35.1 ± 2.10 |

Body weight (g) as assessed in normal aged or sham- and ovariectomy (Ovx)-operated Cnr1/2 double knockout (Cnr1/2^-/-^) and wild type (wt) mice (n = 6 - 8) at the indicated age (days). Values are means ± SD and are obtained from 6-8 animals.

**Table S2. Primers used for amplification of mouse and human osteoclast, osteoblast and adipocyte specific genes.**

| **Primer** | **Forward (5' - 3')** | **Reverse (5' - 3')** | **Probe #** | **Species** |
| --- | --- | --- | --- | --- |
| Tnfrs11a (Rank) | GTGCTGCTCGTTCCACTG | AGATGCTCATAATGCCTCTCC T | 25 | Mouse |
| CTSK | CGAAAAGAGCCTAGCGAACA | TGGGTAGCAGCAGAAACTTG | 18 | Mouse |
| OPN | TCAACATGCACAGAAAAGCTG | TGAATTTGAGTAAGATGTTCTATTTGG | 108 | Mouse |
| CDH11 | GTGCCTGAGAGGTCCAATGT | CCATAGGTGGGATCATCTGC | 110 | Mouse |
| Itgb5 | TTTGCCAAGTTCCAAAGTGA | TCTGTACAGGGGGTTTGAGG | 1 | Mouse |
| Itgb3 | GTGGGAGGGCAGTCCTCT A | CAGGATATCAGGACCCTTGG | 31 | Mouse |
| Nfatc1 | TCCAAAGTCATTTTCGTGGA | TTTGCTTCCATCTCCCAGAC | 50 | Mouse |
| Tcirg1 | CCATATCCCTTTGGCATTGA | GAGAAAGCTCAGGTGGTTCG | 50 | Mouse |
| Runx2 | CCACAAGGACAGAGTCAGATTACA | TGGCTCAGATAGGAGGGGTA | 60 | Mouse |
| Acp5 (TRAcP) | cgtctctgcacagattgcat | aagcgcaaacggtagtaagg | 60 | Mouse |
| AKP2 (ALPL) | aaggcttcttcttgctggtg | gccttaccctcatgatgtcc | 16 | Mouse |
| COL1A1 | ctcctggcaagaatggagat | aatccacgagcaccctga | 79 | Mouse |
| BGLAP | AGACTCCGGCGCTACCTT | CTCGTCACAAGCAGGGTTAAG | 32 | Mouse |
| OPG | atgaacaagtggctgtgctg | cagtttctgggtcataatgcaa | 69 | Mouse |
| RANKL | tgaagacacactacctgactcctg | ccacaatgtgttgcagttcc | 88 | Mouse |
| Cnr1 | gacggtgtttgccttctgtag | gagcatagatgatggggttca | 40 | Mouse |
| Cnr2 | ggcagtgtgaccatgacctt | ggtcaacagcggttagcag | 110 | Mouse |
| PPARγ | GAAAGACAACGGACAAATCACC | GGGGGTGATATGTTTGAACTTG | 7 | Mouse |
| Adrb3 | CAGCCAGCCCTGTTGAAG | GCACCTTCATAGCCATCAAAC | 13 | Mouse |

Abbreviations - Tnfrs11a (Rank), Receptor Activator of Nuclear Factor κB; CTSK, Cathepsin K; OPN, Osteopontin; CDH11, Cadherin 11; Itgb5, integrin beta 5; Itgb3, integrin beta 3; Nfatc1, nuclear factor of activated T cells, cytoplasmic, calcineurin dependent 1; Tcirg1, T-cell immune regulator 1; Runx2, runt related transcription factor 2; Acp5 (TRAcP), tartrate resistant acid phosphatase 5; AKP2 (ALPL), alkaline phosphatase, COL1A1, collagen type I alpha 1 chain; BGLAP, bone gamma-carboxyglutamate protein (Osteocalcin); Tnfsf11 (Rankl), Receptor Activator of Nuclear Factor κB ligand; Cnr1, type 1 cannabinoid receptor (CB1); type 2 cannabinoid receptor (CB2); PPAR peroxisome proliferator activated receptor gamma; Adrb3, adrenoceptor beta 3; CFD, complement factor D (Adipsin).
